# Transforming growth factor-β signaling: from tumor microenvironment to anticancer therapy

**DOI:** 10.37349/etat.2023.00137

**Published:** 2023-04-28

**Authors:** Max Kam-Kwan Chan, Emily Lok-Yiu Chan, Zoey Zeyuan Ji, Alex Siu-Wing Chan, Chunjie Li, Kam-Tong Leung, Ka-Fai To, Patrick Ming-Kuen Tang

**Affiliations:** 1 Department of Anatomical and Cellular Pathology, State Key Laboratory of Translational Oncology, The Chinese University of Hong Kong, Hong Kong 999077, China; 2 Department of Applied Social Sciences, The Hong Kong Polytechnic University, Hong Kong 999077, China; 3 Department of Head and Neck Oncology, West China Hospital of Stomatology, Sichuan University, Chengdu 610041, Sichuan, China; 4 Department of Paediatrics, The Chinese University of Hong Kong, Shatin, Hong Kong 999077, China; Université Paris-Saclay, France

**Keywords:** Transforming growth factor-β, tumor microenvironment, cancer, immunity, cancer immunotherapy

## Abstract

Transforming growth factor-β (TGF-β) signaling is an important pathway for promoting the pathogenesis of inflammatory diseases, including cancer. The roles of TGF-β signaling are heterogeneous and versatile in cancer development and progression, both anticancer and protumoral actions are reported. Interestingly, increasing evidence suggests that TGF-β enhances disease progression and drug resistance via immune-modulatory actions in the tumor microenvironment (TME) of solid tumors. A better understanding of its regulatory mechanisms in the TME at the molecular level can facilitate the development of precision medicine to block the protumoral actions of TGF-β in the TME. Here, the latest information about the regulatory mechanisms and translational research of TGF-β signaling in the TME for therapeutic development had been summarized.

## Introduction

Transforming growth factor-β (TGF-β) cytokine superfamily is composed of highly pleiotropic molecules, including TGF-β, activin, inhibin, bone morphogenetic protein (BMP), etc., which are important for regulating tissue inflammation, fibrosis, cell apoptosis, and proliferation [1]. TGF-β1, 2, and 3 have been defined as three isoforms of TGF-β in mammals [2]. They have been encoded as precursors with 70–80% homology and control basic cell behaviours [3]. However, only TGF-β1 is almost ubiquitous in mammalian tissues. TGF-β1 can be directly activated by different transactivating proteins, such as reactive oxygen species (ROS), plasmin, and acid. Therefore, TGF-β1 is one of the key mediators for executing the physiological and pathological actions of TGF-β signaling [4]. For example, TGF-β is a key activator for tissue fibrosis [5]. It promotes the fibrotic phenotype of immune cells and vascular cells [6, 7]. It also triggers the up-regulation of proto-oncogene tyrosine-protein kinase sarcoma (Src) and brain-specific homeobox/POU domain protein (Pou4f1) for macrophage-myofibroblast transformation (MMT) [8, 9]. Long non-coding RNAs are expressed under the facilitation of TGF-β signaling for enhancing tissue inflammation and fibrosis [10, 11].

Importantly, increasing evidence demonstrates the versatile roles of TGF-β signaling in cancer development and progression. Not only can it suppress carcinogenesis, but it also promotes tumor growth [12, 13] and progression contradictorily [14, 15]. TGF-β signaling is significant in the immunoregulation of the tumor microenvironment (TME) in both adoptive and innate immunity. For instance, TGF-β1 can silence the anticancer activities of T cells and natural killer (NK) cells via transcriptional regulation [15]. It also triggers the generation of cancer-associated fibroblasts via MMT [16]. Both suggest the therapeutic potential of targeting TGF-β signaling in search of immunotherapy against solid tumors. This review systemically summarised the latest updates of TGF-β signaling in TME, as a better understanding of its overall development from basic research to translational works for anticancer therapy.

## Canonical TGF-β pathway

TGF-β superfamily is a group of multifunctional cytokines, including three different mammalian subtypes, TGF-β1, TGF-β2, and TGF-β3 [2]. TGF-β1 is the most common, widely expressed, and well investigated isoform. In fact, the majority of secreted TGF-β are stored in the extracellular matrix (ECM) as latent complexes which act as a reservoir. A latency associated peptide (LAP) and a mature TGF-β undergo non-covalent association to form the small latent complex (SLC) of TGF-β. Often, the LAP in the SLC covalently binds to a binding protein, forming the large latent complex (LLC) [17]. Despite its abundance, downstream signaling of the pathway is prohibited without ligand activation. Multiple mediators are responsible for the release and activation of LAP-TGF-β. Integrin, thrombospondin, matrix metalloproteinases (MMPs), plasma kallikrein, and plasmin are examples of activators [18]. The latency of TGF-β release and activation provides a means to regulate the bioavailability of TGF-β locally. Local changes in the ECM under pathological conditions are detected and responded to the release of TGF-β from the latent complexes to facilitate tissue repair.

Once activated, the TGF-β ligand regulates cellular processes by binding to TGF-β type I receptors (TβRI) and TβRII, both of which are transmembrane protein serine/threonine kinases [19]. After binding of TGF-β to TβRII, it will recruit TβRI and form a functional heterologous complex. Activation of receptor-associated mothers against decapentaplegics (R-Smad), namely mothers against decapentaplegics 1 (Smad1), 2, 3, 5, and 8, is triggered when TβRII transphosphorylases the glutamine synthetase (GS) domain of TβRI which results in the activation of TβRI kinase. Then R-Smads and common-mediator Smad (Co-Smad) combine with Smad4 to form a heterotrimeric complex which later translocates into the nucleus for regulation of transcriptional activity [20, 21]. The Smad complex cooperates with other transcription factors, chromatin binding proteins, transcription co-activators, and co-repressors at the genome level [22].

**Figure 1.**
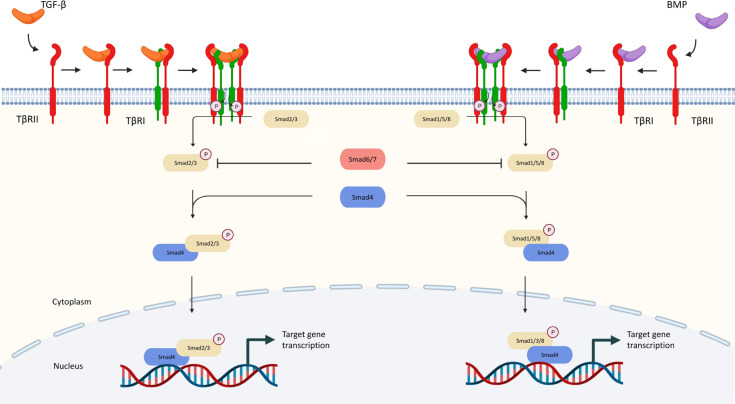
Canonical pathway of TGF-β signaling. TGF-β communicates through the Smad and non-Smad signaling pathways. TGF-β interacts with TβRII in the Smad pathway, which phosphorylates TβRI and activates Smad2 and Smad3 (R-Smads). Activated Smad2/3 produces R-Smads-Co-Smad complexes with Smad4 (Co-Smad), which interact with several transcription factors and co-activators and control the transcription of target genes. BMPs bind to homomeric type II receptors, which transphosphorylate homomeric type receptorsors to generate both Smad-dependent and independent signaling. Phosphorylated R-Smad (Smad1, 5, or 8) binds with Smad4 and co-translocates into the nucleus during Smad-dependent signaling. P: phosphorylation

In particular, Smad2 and Smad3 are phosphorylated by activated TGF-β receptors, while Smad1, 5, and 8 are downstream of BMP receptors [22]. Additionally, there is a subfamily of inhibitory Smads (I-Smads), with members including Smad6 and Smad7. I-Smads block the signaling through multiple mechanisms which serve as essential negative feedback for preventing the hyperactivation of TGF-β signaling [23]. For example, Smad6 binds to Smad1 and thereby prevents its binding to Smad4. Smad7 blocks the phosphorylation and activation of Smad2/3 by competing with their binding to TβRI [5]. The canonical TGF-β pathways are summarized in Figure 1.

## Non-canonical TGF-β pathway

**Figure 2.**
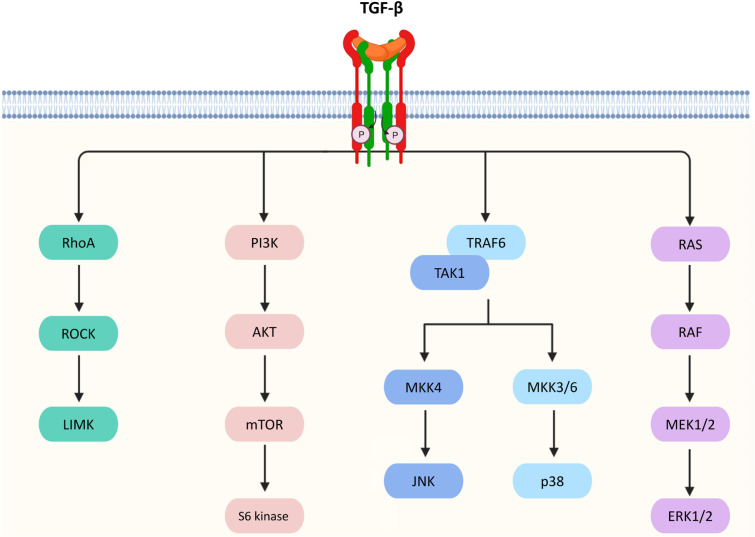
Non-canonical pathways of TGF-β signaling. TGF-β triggers Ras homolog gene family member A (RhoA)/Rho-associated protein kinase (ROCK) and then activates the p-Lin-11/Isl-1/Mec-3 kinases (LIMK) to mediate epithelial mesenchymal transition (EMT). TGF-β signaling can also activate protein kinase B (AKT) via phosphoinositide 3-kinase (PI3K) for regulating translational responses though the mammalian target of rapamycin (mTOR) and S6 kinase. In addition, TGF-β activates tumor necrosis factor receptor-associated factor 6 (TRAF6) and TGF-β-activated kinase 1 (TAK1), which facilitates the activation of p38 mitogen-activated protein kinase (MAPK) and c-Jun N-terminal kinase (JNK)/MAPK via the MAPK kinase 3/6 (MKK3/6) and MKK4 pathways, respectively, resulting in the control of cell death, proliferation, metastasis, growth, and angiogenesis. Furthermore, TGF-β can interact with Ras, which then triggers the gene regulatory actions via the rapidly accelerated fibrosarcoma (RAF), MAPK kinase 1/2 (MEK1/2), and extracellular signal-regulated kinases 1 and 2 (ERK1/2) to regulate cell proliferation, apoptosis, and differentiation

Besides the canonical pathway, TGF-β achieves its biological actions via a number of non-canonical pathways in a Smad-independent manner which is listed as follows and summarized in Figure 2.

### ERK

TGF-β activated TβRI phosphorylates Src homology and collagen A (ShcA) protein on the serine and tyrosine residues on the receptors [24]. The ShcA protein acts as a docking site for binding of the Son of sevenless (Sos) protein and growth factor receptor binding protein 2 (GRB2) [25]. The formation of a Shc/GRB2/Sos complex converts Ras into its guanosine triphosphate (GTP)-bound active form. It phosphorylates c-RAF for the activation of MEK1/2 kinase, which serves to regulate cell proliferation, apoptosis, and differentiation [26, 27].

### JNK/p38

TGF-β activates JNK and p38 MAPK through TRAF6 and TAK1 [28]. TRAF6 undergoes K63-linked polyubiquitination to activate TAK1, which promotes MKK3/6 and MKK4-mediated activation of p38 MAPK and JNK/MAPK respectively [29]. The p38 MAPK and JNK/MAPK execute TGF-β-mediated regulatory actions via activating transcription factor 2 (ATF2) [30] or EMT [31].

### Rho-like GTP hydrolases

RhoA GTP hydrolase (GTPase) involves in TGF-β-mediated cytoskeletal regulation and stabilization of adhesion junctions, abnormalities in these processes may cause EMT [32]. RhoA is activated under the action of TGF-β, resulting in higher levels of RhoA binding to GTP [33]. This stimulates the formation of actin stress fibres [34]. GTP-RhoA also turns on ROCK and then activates LIMK, which inactivates an actin depolymerizing factor (cofilin). At the tight junctions of the epithelial cells, the activity of RhoA is directly regulated by TGF-β [35] to mediate EMT for invasion of mesenchymal cells and endothelial activation [36].

### PI3K/AKT

TGF-β also activates PI3K to promote the activation of AKT via mTOR complex 2 (mTORC2) [37], which regulates cell survival, migration, and growth. Downstream of AKT, S6 kinase 1, can be triggered by activated mTOR. However, AKT can cooperate with Smad3 to inhibit its transcription-mediated activity, thereby attenuating TGF-β-induced apoptosis [38]. AKT also inhibits a tumor suppressor and glycogen synthase kinase-3β (GSK-3β) protein forkhead box O (FoxO) transcription factor to prevent the Smad-mediated growth arrest and metastasis [39, 40]. Interestingly, RAS and TGF-β signaling induces the secretion of platelet-derived growth factor (PDGF) and its autocrine activity by upregulating PDGF receptors. It results in the activation of PI3K and the accumulation of nuclear β-catenin. EMT is promoted [41–43].

### Other TGF-β-dependent mechanisms

The Abelson tyrosine kinases (c-Abl) has been identified as an important mediator in TGF-β signaling for tissue fibrosis and fibroblast formation [44]. Activated c-Abl mediates the TGF-β-induced morphological changes, extracellular matrix gene expression, and fibroblasts’ proliferations [44]. Besides, protein kinase A (PKA) has been discovered to take part in TGF-β-mediated gene transcription without cyclic adenosine monophosphate’s (cAMP’s) involvement in pancreatic acinar cells [45]. PKA can be activated by a Smad3/Smad4 complex by interacting with its regulatory subunits, which is essential in CREB-dependent growth inhibition [25].

## Signaling crosstalk with other pathways

**Figure 3.**
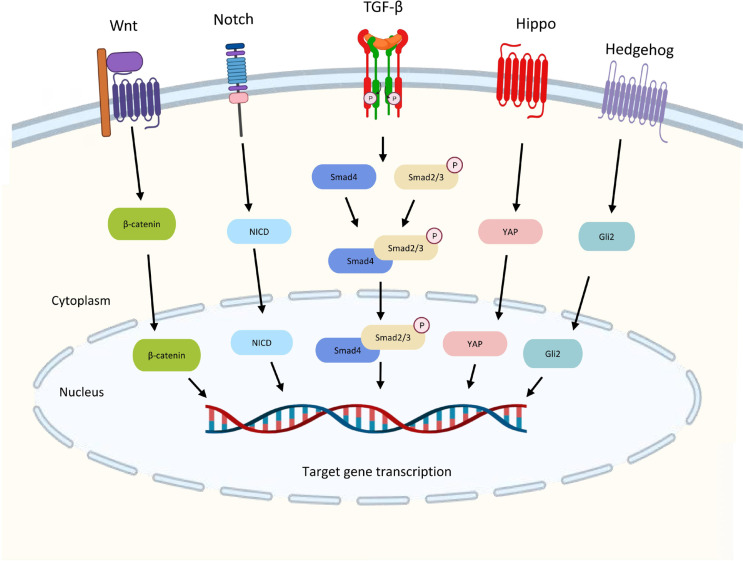
Crosstalk between the TGF-β pathway and other signaling pathways. TGF-β and Wnt signaling interact to activate the transcription of gastrin by the complex of Smad3 and β-catenin to promote gastrointestinal cancer. Downstream transcription factor Yes-associated protein (YAP) in Hippo signaling can also form a complex with Smad3 to drive malignant development. The Notch intracellular domain (NICD) from Notch signaling is required for TGF-β-induced EMT. High Gli2 levels in hedgehog accelerate TGF-β/Smad3-induced EMT and tumor growth. Wnt: wingless-related integration; Gli2: glioma-associated oncogene 2

The TGF-β pathway is integrated into the intracellular signaling network through crosstalk with other signaling pathways, and these crosstalk activities play crucial roles in the regulation of a variety of biological responses. The TGF-β signaling crosstalk with other pathways are summarized in Figure 3.

TGF-β and Wnt signaling interact to activate the transcription of the Wnt target gene for gastrin, a promoter of gastrointestinal cancer, suggesting that the two may work together to induce carcinogenesis. [46]. The downstream transcription factor YAP in Hippo signaling may also crosstalk with TGF-β signaling. YAP-transcriptional enhancer factor domain family member 4 (TEAD4)-Smad3-p300 complex forms at the connective tissue growth factors (CTGF) promoter in mesothelioma cells increasing the expression of CTGF for driving malignant development [47]. Notch signaling is required for TGF-β-induced EMT and cell differentiation in keratinocytes, normal murine mammary gland epithelial cells, and primary kidney tubular epithelial cells [48]. High Gli2 levels are noted in various malignant tumor cells, including melanoma, breast cancer, glioblastoma, and ovarian cancer. High TGF-β levels also exhibit in these samples [49]. High Gli2 expression is related to decreased E-cadherin’s expression and enhanced tumor cell invasion which may accelerate TGF-β-induced EMT and tumor growth [50].

## TGF-β signaling in cancer cells

In normal and premalignant cells, TGF-β strengthens homeostasis and inhibits tumor progression by inducing cell arrest, differentiation, and apoptosis, directly or indirectly through its impact on the matrix (including inhibition of inflammation and matrix-derived mitogen).

### Inhibitory roles in cancer formation

TGF-β plays a vital role in cancer, acting as a tumor suppressor in the initial stages of tumorigenesis. TGF-β primarily reduces cell proliferation by activating cyclin-dependent kinase (CDK) inhibitors and suppressing the expression of cellular myelocytomatosis oncogene (C-MYC) [51]. TGF-β inhibits cell proliferation in neurons, epithelial cells, and hematopoietic cells via targeting CDK and its inhibitor (CDK-I) [17]. CDK involves in the regulation of cell cycle progression after the Gap 1 phase (G1) in cell proliferation [52]. TGF-β also stimulates the expression of CDK-I inhibitors, such as P15^INK4B^, P21^CIP1^, and P27^KIP1^ [53]. These inhibitors block the cyclin-CDK complex, resulting in the arrest of the cell cycle in the G1 phase [54].

P15 suppresses cell cycle progression in the latter stages of G1 by primarily inhibiting the communication between CDK4/6 and cyclin D. P21 directly binds to the proliferating cell nuclear antigen (PCNA) and cyclin E-CDK2 complex to inhibit PCNA-dependent DNA replication and CDK’s activity respectively. Moreover, P27 interacts with the cyclin E-CDK2 complex by removing it from the cyclin D-CDK4 complex [54]. Inactivating these CDK complexes inhibits the phosphorylation of retinoblastoma protein (pRb). Cell cycle progression requires the phosphorylation of pRb, and its suppression prevents G1 cells from entering the S phase [52]. Meanwhile, TGF-β suppresses cell growth by down-regulating the expression of the C-MYC oncogene that promotes cell division and permits cells to reproduce uncontrollably [52]. Besides, TGF-β reduces the expression of inhibitor of DNA binding 1 (Id1), Id2, and Id3 [55, 56]. The Id-protein family consists of nuclear factors related to the transition from G1 to S phase in the cell cycle. Cell growth is decelerated when Id proteins are inhibited. TGF-β is also associated with an antiproliferative response via the Smad-dependent pathway [53]. Inhibition of P70 S6 kinase by TGF-β through the protein phosphatase 2A (PP2A) pathway will lead to cell cycle arrest in the G1 phase [57].

The Smad-dependent and Smad-independent pathways are the two major mechanisms by which TGF-β induces apoptosis in a variety of cell types, hence suppressing tumor development. The lack of Smad4 accelerates the malignant evolution of tumors caused by carcinogenic stimuli [58]. Pro-apoptotic proteins are involved in the Smad-dependent pathways, such as Src homologous inositol phosphatase (SHIP), death-associated protein kinase (DAPK), and TGF-β-induced early gene 1 (*TIEG1*). By coupling Smads to mitochondrial-based pro-apoptotic processes, DAPK facilitates TGF-β-dependent apoptosis and stimulates the release of cytochrome C. SHIP interferes with the PI3K-AKT signaling pathway, leading to cell death [59].

TIEG1 causes oxidative stress by generating ROS [60]. Cell apoptosis is promoted, thereby inhibiting tumor growth. The stress activate protein kinase (SAPK)/JNK signaling pathway induced by deleted in pancreatic cancer 4 (DPC4) is also involved in TGF-β signaling, leading to cell apoptosis [61]. TGF-β-mediated apoptosis is required for caspase activation in the TGF-β-independent pathway. TGF-β suppresses the production of anti-apoptotic genes such as B cell lymphoma-2 (*Bcl-2*), Bcl-extra large (*Bcl-xL*), and X-linked apoptotic inhibitor (*XLAP*) [62]. Furthermore, TGF-β stimulates the production of numerous pro-apoptotic genes, including *caspase 3*, *caspase 8*, and Bcl-2 interaction killer (*BIK*). Death domain-associated protein (DAXX) is a protein related to the FAS receptor and is also involved in TGF-β-induced apoptosis by activating JNK [63].

### Pathogenic roles in cancer progression

In many cancers, there are mutations or deletions of TGF-β receptors or Smads in the TGF-β pathway, leading to the inactivation or disturbance of the signaling [58]. The EMT of tumor cells is essential for cancer metastasis. It is required for the advancement of fibrosis, cancer, and embryonic development [36]. TGF-β-induced EMT increases tumor invasion and dissemination during cancer growth and progression by releasing and transporting tumor cells into the environment. It controls the transcription of E-cadherin, N-cadherin, Snail, and vimentin in multiple malignancies [64]. In terms of cytoskeletal remodeling, consistent expression of the myofibroblast is supported by TGF-β and adhesion-mediated signaling. After EMT, epithelial cells lose their polarity, tight junctions, and intercellular adhesion, gaining the potential to migrate [65]. The capacity of tumor cells to metastasize is enhanced by reducing cell adhesion [66].

Angiogenesis during tumor growth is critical since tumor cells need an increased supply of nutrients and oxygen for proliferation and invasion [67]. Endothelial cells highly participate in angiogenesis, which indicates increased cell proliferation, migration, and invasion in the neovascular system [68]. TGF-β signaling modulates the activity of endothelial cells in a complex manner. TGF-β1 promotes the release of pro-angiogenic growth factors such as vascular endothelial growth factors (VEGF) and CTGF. VEGF induces endothelial cells to assemble capillaries for generating and maintaining tumor angiogenesis whereas CTGF enhances capillary density, thereby accelerating cancer progression [69]. The anti-cancer effects of TGF-β are often lost in cancers. Many studies have analyzed the role of TGF-β in the development of resistance to anti-cancer drugs, but its underlying mechanism is still unclear [70]. For example, TGF-β1 promotes oxaliplatin resistance in colorectal cancer patients through EMT [71].

## TGF-β signaling in TME

**Figure 4.**
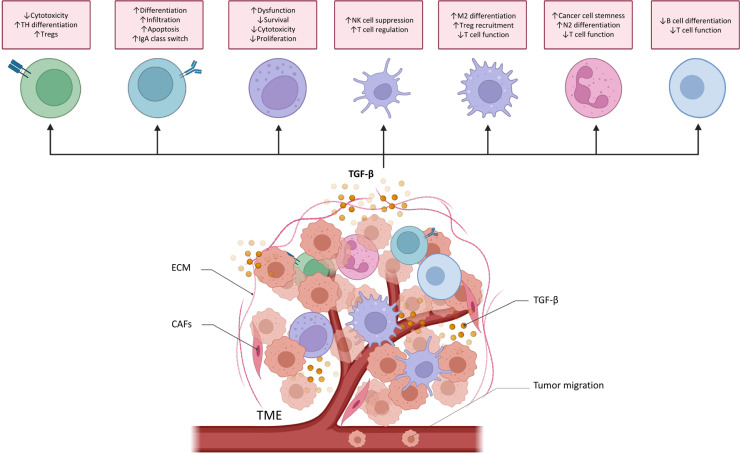
TGF-β functions in cancer immune surveillance. TGF-β directly promotes T helper 17 (TH17) cell differentiation into forkhead/winged helix transcriptional factor P3 (FoxP3^+^) regulatory T cells (Tregs) and suppresses cytotoxic CD8^+^ T cell development to drive cancer progression. TGF-β regulates the differentiation, proliferation, and apoptosis of B cells. It increases the activity and infiltration of tumor-educated B cells in order to inhibit T cells and NK cells proliferation and their antitumor effects. It also controls the class switch from immunoglobulin M (IgM) to IgA in B cells. TGF-β causes NK cells dysfunction and reduces the proliferation and survival of NK cells. TGF-β also inhibits the production of cytotoxic cytokines. TGF-β alters DCs’ function, thereby inhibiting NK cells and promoting T cell differentiating into Treg. TGF-β induces the M2 differentiation of macrophage and then increases the recruitment of Treg cell. TGF-β secreted by tumor-associated macrophage (TAM) can also suppress the cytotoxicity of T cells. TGF-β induces the N2 differentiation of neutrophils. TGF-β secreted by tumor associated neutrophils (TANs) increases cancer cell stemness and attenuates T cell response. Myeloid-derived suppressor cells (MDSCs)-derived TGF-β inhibits B cell differentiation and T cell function, helping cancer cells to avoid immune surveillance. ↑: increases; ↓: decreases; DCs: dendritic cells

TGF-β plays an important role in TME. It regulates the infiltration of inflammatory cells, inhibits immune surveillance, and promotes the recruitment of macrophages and monocytes, thereby promoting the progression of metastasis [16, 72–74]. Therefore, TGF-β is essential for the immune regulation of TME and different immune cells (Figure 4).

### Protumoral effect of TGF-β signaling in TME

TGF-β represses the antitumor effect of T-cells and their proliferation and recruitment [75]. TGF-β inhibits cytotoxic T cell proliferation by impeding their cell cycles or downregulating interleukin-2 (IL-2) receptors. TGF-β inhibits CD4^+^ T-box expressed in T cells (T-bet) and promotes Tregs through Smad3 and NFAT [76, 77]. Treg cell differentiation requires mTORC1 downregulation and TGF-β transcriptional reprogramming [78]. TGF-β reduces the antitumor activity of cytotoxic T cell by converting naive CD4^+^ T cells into TH17 and inducible Treg (iTreg) [79]. TGF-β lowers the level of cytotoxic T-lymphocyte (CTL) perforin, granzyme A and B, FAS, and interferon-γ (IFN-γ). TGF-β also inhibits Smad2/anaplastic lymphoma kinase 5 (ALK5)-mediated CD8^+^ T cell tumor invasion. Special AT-rich sequence binding protein 1’s (SATB1’s) TGF-β suppression increases T follicular helper (Tfh) proliferation [80]. In patients with liver gastrointestinal cancer cells and gastric cancer [80, 81], TGF-β upregulation is associated with low numbers of CD1a^+^ and CD83^+^ DCs in the tumor stroma. TGF-β upregulates the expression of the first member of the B7 family (B7H1) and glucocorticoid-induced tumor necrosis factor receptor-related protein ligand (GITRL) in DCs, which substantially promotes the formation of Treg in lung cancer [82]. TGF-β1 released by cancer stem cells is involved in the generation of DC-mediated immunotolerance in liver cancer [83]. In pancreatic cancer, TGF-β contributes to the T lymphocyte-driven inhibition of DC antitumor immunity [84]. *In vitro* research indicates that co-culturing mature DCs with melanoma cells decreases TGF-β synthesis, hence promoting T-cell proliferation [85]. In a canine transferrable tumor model, tumor-derived TGF-β impairs DCs’ maturation, which may be reversed by IL-6 through Smad2/3 nuclear translocation interference [86].

TGF-β pathway increases the expression of CD39 and CD73 in MDSCs by activating hypoxia-inducible factor-1 (HIF-1) and hence suppressing T cells’ and NK cells’ activity in non-small-cell lung cancer (NSCLC) [86]. TGF-β involves in tumor-induced dysfunction of NK cells in breast cancer [87]. TGF-β converts NK cells into intermediate type 1 innate lymphoid cells (intILCs) to limit innate immune surveillance in the TME, this innate lymphoid cell (ILC)-like phenotype loses the metastasis-restraining function of NK cells [88]. TGF-β1 promotes tumor-associated NK cells to become non-cytotoxic in prostate cancer via weakening NK group 2D (NKG2D) and DNAX accessory molecule 1 (DNAM-1) [89]. Loss of circulating NK cells with activating receptors [NK protein 30 (NKp30), NKp46, NKG2D, and DNAM-1] is linked to gastric cancer progression [90]. TGF-β1 also suppresses NK cell development in lung cancer and melanoma via a Smad3/erythroid-derived 4 binding protein 4 (E4BP4) axis [15]. TGF-β1 produced from platelets increases neutrophil recruitment to tumors and enhances metastasis in tumor-bearing mice [91]. TGF-β represses the N1 anticancer phenotype of neutrophils [92]. Sagiv et al. [93] showed that TGF-β therapy causes N1-like high-density neutrophils to become N2-like low-density neutrophils in the TME. TAN also releases TGF-β2 to stimulate microRNA-301-3p (miR-301-3p)-related stem cell features in oncogenic hepatocytes to increase angiogenesis in hepatocellular carcinoma (HCC) [94]. TGF-β signaling in neutrophils reduces T cells’ response and produce a metastatic environment in colorectal cancer (CRC) [95].

Besides immune cells, cancer associated fibroblasts (CAFs) induces EMT in human NSCLC cells through Smad3 activation [96]. CAFs induce prostate cancer cell proliferation, migration, and treatment resistance through TGF-β [96, 97]. Evidence shows that CAF-derived TGF-β enhances lung adenocarcinoma development [98]. TGF-β signaling enhances the invasiveness of cutaneous squamous cell carcinoma (SCC) while promoting EMT [99]. In addition, CAFs induce malignancy by secreting TGF-β1 [100] and activating Smad2/3/4-mediated homeobox transcript antisense RNA (HOTAIR) transcription [100]. TGF-β is known to promote myofibroblast differentiation and contractility. Functional markers expression in human fibroblasts, including alpha smooth muscle actin (α-SMA), tenascin-C (TNC), prolyl 4 hydroxylase (P4H), and fibroblast activation protein (FAP), is also supported [101]. Noticeably, TGF-β1 accelerates oral SCC invasion by activating autophagy in CAFs [102]. TGF-β signaling downregulates isocitrate dehydrogenase 1 (IDH1) in fibroblasts to increase glutamine metabolization and tumor development [103] whereas TGF-β1 in CAF-derived exosomes activates the Smad pathway [104]. Paracrine TGF-β1 from CAFs promotes breast cancer cell EMT [105]. Cancer cell-derived TGF-β enhances CAF’s fibrogenic activation and breast cancer cell intravasation and extravasation [106]. Exosome-harboured TGF-β enhances fibroblast differentiation into CAFs [107]. Recently, the research found that TAM might trans-differentiate into cancer-associated fibroblasts in lung carcinoma through a TGF-β/Smad3-dependent pathway known as “macrophage-myofibroblast transition”, as shown by single-cell RNA-sequencing analyses [16]. TAMs also promote *de novo* neurogenesis by “macrophage to neuron-like cell transition”. It exhibits neuronal phenotypes and expresses pain receptor which associates with cancer-driven nocifensive behaviors [73].

### Anticancer effect of TGF-β signaling in TME

In contrast, limited anticancer effects of TGF-β signaling have been identified in TME. For example, TGF-β regulates B cell differentiation, proliferation, and death [108]. It is also one of the cytokines that control B cell IgM-to-IgA class switching. In Peyer’s patches, IgA switching requires B cell-DC contact because DC-expressed integrin the alpha v beta 8 integrin (αvβ8) triggers TGF-β to increase B cell maturation and IgA synthesis. TGF-β1-induced TGF-MDSCs show reduced immunosuppressive effects, improved antigen-presenting capacity, and higher cancer-killing capability [109]. Smad4 can impede cell conversion by inhibiting the non-canonical TGF-β pathway in NK cells [110].

## Clinical implications of TGF-β signaling in cancer therapy

**Figure 5.**
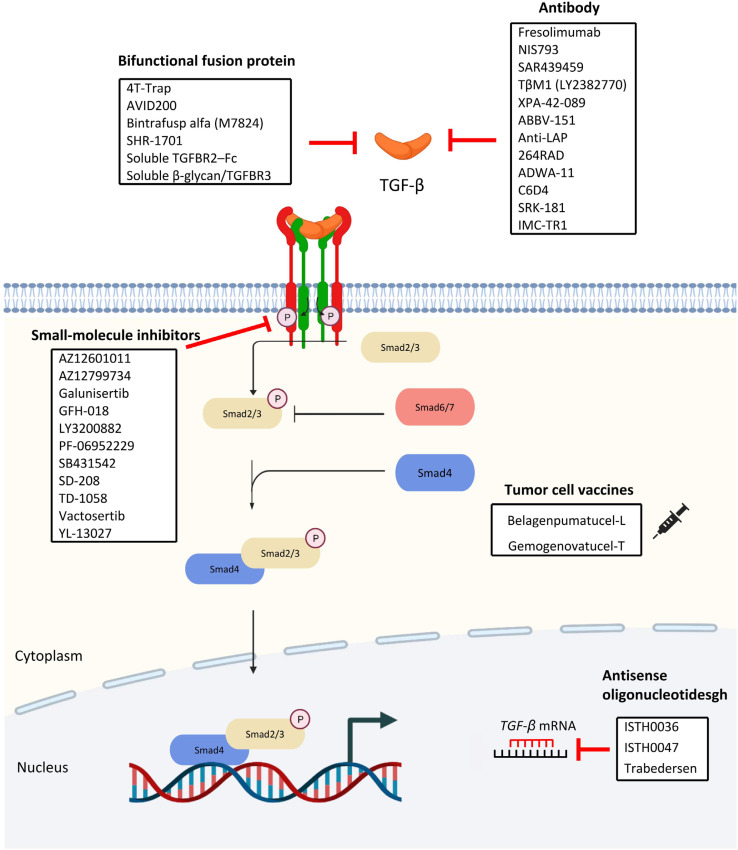
Overview of TGF-β signaling therapeutic targets

Due to its potentials in promoting tumor growth, the TGF-β-signaling pathway provides prospective options for targeted treatment. To investigate the effects of TGF, knockout mice of TGF-β associated proteins have been studied as summarized in Table 1. In addition, following the completion of experiments on animals and the establishment of targets, clinical research on a variety of medications aiming at distinct components in the TGF-β pathway has been conducted. Some of which are currently being produced and evaluated (Figure 5).

**Table 1.** Overview of knockout or knock-in mouse models of TGF-β-associated protein

**Knockout mouse model**	**Phenotype**	**Reference**
Smad5	Embryonic fatal; faulty vascular development, ventral closure, cardiac development, and craniofacial development; aberrant heart looping and embryonic turning	[111, 112]
Smad3	A reduced size compared to littermate controls	[15, 113]
Smad7	Body sizes were smaller than wild-type mice	[114]
Activin βA	Newborn fatality; cranial defects (cleft palate and loss of whiskers, upper incisors, lower incisors, and lower molars)	[115, 116]
Activin βB	Large litters but delayed parturition; breastfeeding issues; birth abnormalities in eyelid closure	[117]
Activin βC	No noticeable abnormalities; viable	[118]
Activin βE	No noticeable abnormalities; viable	[118]
Activin βB knockin to the activin βA locus	Reversal of activin A-deficient neonatal mortality. Defects in the development of the hair, gonads, external genitalia, and somatic growth	[119]
Growth differentiation factor 9 (GDF-9)	Viable; female infertility; one-layer primary follicle stage halt of folliculogenesis	[120, 121]
Inhibin α	Female infertility; secondary male infertility; granulosa/Sertoli cell and adrenal tumors; cachexia-like condition	[122]
Activin receptor type II	Infertility in females is caused by a folliculogenesis abnormality; delayed fertility in males; undersized gonads; 25% of mice die at birth owing to mandible deformities	[116]
FKBP12	Due to cardiomyopathy and neural tube abnormalities, the majority of mice perish between embryonic day 14.5 (E14.5) and delivery	[123]
Follistatin	Neonatal fatality; craniofacial abnormalities, development retardation, and skin abnormalities	[111]

### Antibodies

Over the years, antibodies targeting the three human TGF-β isoforms TGF-β1, TGF-β2, and TGF-β3 have been developed and studied.

Fresolimumab (GC1008) is a monoclonal IgG4 antibody designed to reduce effector immune cell function through the neutralization of TGF-β1, TGF-β2, and TGF-β3, it was clinically investigated as monotherapy or in combination with radiotherapy in renal cell carcinoma, pleural mesothelioma, NSCLC, metastatic breast cancer and glioma phase I and II clinical trials in which it showed promising antitumor activities with minimal toxicities [124].

NIS793, another human IgG2 monoclonal antibody that is designed to neutralize the pan TGF-β ligands, was well-tolerated in phase I clinical trial. Currently, it is being investigated in phase I to III clinical trials, working in combination with chemotherapeutic drugs or immune cell checkpoint inhibitors [125].

With the success seen in fresolimumab, SAR439459 was developed with an altered Fc region. Such improvement led to the reinvigoration of NK and T cells and sensitisation of programmed cell death protein 1 (PD-1) blockade on T cells in preclinical settings [126]. Currently, SAR439459 is being investigated in a phase I clinical trial in combination with cemiplimab in patients with advanced solid malignancies.

TβM1(LY2382770) is a highly selective TGF-β1 neutralizing antibody that was previously assessed in a phase I dose-escalation study of metastatic cancer. Although TβM1 showed no sign of toxicity in the study, no clinical benefits could be observed due to increased tumor markers after administration [127]. TβM1 development was put to an end after this study.

XPA-42-089 is an α-pan-TGF-β antibody which is designed to block TGF-β1 and TGF-β2. A preclinical study showed that XPA-42-089-mediated TGF-β blockade led to the restoration of anti-tumor immunity in a PD-1 resistant immunosuppressive environment [125].

Unlike the aforementioned antibodies, ABBV-151 is a monoclonal antibody specifically designed to block the GARP-TGF-β1 complex, subsequently blocking the release of TGF-β1. It is currently being investigated in phase I clinical trial in combination with an anti-PD-1 antibody budigalimab in patients with metastatic solid tumor [128].

To target the latent complex that retains inactive TGF-β, anti-LAP was developed. LAP is encoded by the *TGF-β1* gene and cleaved by furin to reassemble with TGF-β, which then be stored on the cell surface as a latent complex. LAP is upregulated on Foxp3^+^ regulatory T cells which suppress anti-tumor immunity in the cancer microenvironment. In a preclinical study, anti-LAP treatment enhanced adaptive immune response against cancer reduced CD103^+^ regulatory T cell numbers and promoted CD8^+^ T effector cells antitumor activity [129].

264RAD targets the TGF-β pathway through αvβ6 and αvβ8 integrin inhibition which is distinct from the mentioned antibodies. 264RAD treatment showed improvements on xenograft mice survival, and reduction in tumor proliferation, growth, and migration in breast cancer and pancreatic ductal adenocarcinoma preclinical models [130].

C6D4 is an αVβ8 neutralizing antibody that inhibited colonic carcinoma and prostatic tumor growth in a preclinical study published recently [131]. αVβ8 is crucial to the activation of the TGF-β pathway and is frequently overexpressed in a variety of tumor cells. It was reported that C6D4 treatment increased cytotoxic T cell anti-tumor activity and promoted the recruitment of immune cells to tumor centers. Particularly, various cancers expressing a high level of αVβ8 are not usually associated with high programmed cell death ligand 1 (PD-L1) expression, suggesting anti-αVβ8 is a potential immunotherapeutic agent working independently of the PD-1/PD-L1 pathway.

Interestingly, another αVβ8 antibody, ADWA-11 also inhibited tumor growth in various human cancers in combination with radiotherapy or immune checkpoint inhibitors in another preclinical study [132]. Congruent with the previously mentioned study, ADWA-11 treatment restored cytotoxic T cell’s function *in vitro*, solidifying that αVβ8 could potentially serve as an immunotherapy target.

SRK-181 is a monoclonal antibody designed to prevent latent TGF-β1 activation. Before the phase I clinical trial, SRK-181 demonstrated its ability to overcome anti-PD-1 resistance in mice xenograft models. Currently, SRK-181 treatment was well-tolerated in test subjects and led to tumor regression in an ongoing phase I clinical trial [133].

Finally, LY3022859, an anti-TβRII IgG1 monoclonal antibody, was one of the first anti-TGF-β antibodies to be clinically evaluated [134]. Unfortunately, the clinical trial was halted prematurely because the maximum tolerated dose could not be determined during the study. A dose higher than 25 mg was deemed unsafe due to uncontrollable cytokine release.

### Antisense oligonucleotides

Oligonucleotide-based gene regulators, such as antisense oligonucleotides (ASOs) and small interfering RNAs (siRNAs) targeting messenger RNA (mRNA), are potential agents for the treatment of disorders resistant to conventional small molecule medications. These gene regulators can be built easily based on the target sequence, without taking into account the complicated protein structures and give knock-down of targets [135]. As prospective therapeutic agents for the treatment of fibrosis in various organs, such as the heart, kidney, and gut, ASOs and siRNAs targeting TGF-β and the related downstream factors have been deployed [136–139].

ISTH0036 is a 14-mer modified antisense oligodeoxynucleotide for targeting *TGF-β2* RNA. The first-in-human phase I study of ISTH0036 in patients with primary open-angle glaucoma showed potential clinical efficacy. The results showed that a single dose of ISTH0036 which selectively inhibited *TGF-β2* mRNA was safe [140].

ISTH0047 or ISTH1047 significantly reduces TGF-β1 or TGF-β2 expression in various human and mouse glioma cells. ISTH0047-mediated TGF-β2 inhibition reduces the highly invasive phenotype of LN-229 gliomas and eliminates the expression of phosphorylated Smad 2 (pSmad2) [141]. ISTH0047 elevates the expression of CD86 in TAMs and inhibits lung metastasis in syngeneic orthotopic renal cell carcinoma and breast cancer tumor models by reducing the expression of TGF-β2 [142].

Trabedersen (AP 12009) specifically and effectively inhibits TGF-β2 expression in patient-derived malignant glioma cells and peripheral blood mononuclear cells [143]. Trabedersen inhibits the migration of pancreatic carcinoma, glioma, and malignant melanoma cells by interfering with the autocrine loop of TGF-β2 secretion [144]. In a randomized and controlled phase IIb study, trabedersen transiently reduced TGF-β2 levels to reverse TGF-β2—mediated immunosuppression, and increased patients’ survival rate [145].

### Bifunctional fusion proteins

Bifunctional fusion proteins yield efficacy by binding to two distinct molecules. A subset of bifunctional fusion proteins consists of a unique class of antibody-cytokine fusions that may enhance anticancer immunity [146].

4T-Trap is a bispecific receptor decoy that efficiently binds to CD4^+^ T cells and inhibits Th cell TGF-β signaling in tumor-draining lymph nodes as revealed by surface plasmon resonance. 4T-Trap blocks TGF-β signaling to increase Th2 cell differentiation and induces tumor cell death [147].

AVID200 is an engineered receptor ectodomain-based trap that neutralizes TGF-β1 and TGF-β3. This protein trap inhibits TGF-β1—induced p57Kip2 expression and blocks the activation of Smad2 by neutralizing recombinant TGF-β1 (rTGF-β1) and rTGF-β3 in myelofibrosis progenitor cells. AVID200 inhibits the expression of pSTATS3 in mesenchymal stromal cells treated with rTGF-β1 [148].

Bintrafusp alfa (M7824) simultaneously targets TGF-β and PD-L1 in breast carcinoma cells. M7824 reduces the expression of TGF-β and blocks PD-L1 on CD8^+^ T cells to activate their anti-tumor responses in murine models of human solid carcinomas [149]. M7824 prevents fibronectin and vimentin initiation in PC-9 and A549 cells by preventing Smad2 and Smad3 activation in response to TGF-β1 [150]. M7824 blocks PD-L1-dependent immunosuppression in NSCLC. It can be safely managed and has antitumor activity in patients with advanced solid tumors [151].

SHR-1701, a bispecific fusion protein, increases IFN-γ and Ki-67 expression in peripheral CD8^+^ T cells in patients with impaired lymphocyte recovery by blocking the TGF-β/TβR downstream pSmad2 signaling pathway and rescues the downstream phosphorylated Akt (pAkt) pathway of PD-1/PD-L1 [152]. In phase I trial, SHR-1701 showed safety and antitumor activity in patients with recurrent or metastatic cervical cancer. It is observed that the level of pSmad2 in immune cells might predict the response to SHR-1701 [153].

Soluble TβRII-Fc (sTβRII-Fc) binds with TGF-β1 and inhibits TGF-β1-dependent Smad3 phosphorylation *in vitro* [154]. An Adenovirus 5 (Ad5)-based oncolytic virus expressing sTβRII-Fc fusion protein (Ad.sTβRFc) and a 01/07 based adenoviral vector expressing the soluble form of TβRII fused with human *Fc IgG1* gene (mhTERTAd.sTβRFc) vectors can induce the secretion of TβRII-Fc into the tumor-bone microenvironment. Undesirable TGF-β signaling in breast tumor cells, osteoclasts, and osteoblasts is observed to be inhibited [155]. sTβRII-Fc protein binds with TGF-β1 and inhibits TGF-β-stimulated p38 MAPK to interfere with breast cancer invasion and metastasis [156].

Soluble β-glycan/TβRIII mainly activates TGF-β2-Smad signaling and the expression of TβRIII reduces the anti-tumor function of TGF-β2 in clear-cell renal cell carcinoma. Downregulation of TβRIII reduces TGF-β-independent cell migration by increasing lamellipodia formation via focal adhesion kinase (FAK)-PI3K signaling [157].

### Small molecules inhibitors

TGF-β1 and its receptor, TβRI are essential components of the TGF-β signaling pathway important for immune response. Studies revealed an increased level of TGF-β1 and TβRI in different cancer samples, such as gastric cancer and hepatocarcinoma. Over-expression of TβRI in tumor and its response to TGF-β1 would lead to dysregulation of cell growth and proliferation, which was found to have a close association with malignancies development [158]. However, the complete knockout of TβRI in mice would result in life-threatening chondrodysplasia [159]. Thus, inhibition but not the elimination of TβRI can be a possible direction of immunotherapeutic research. In the following section, small molecules inhibitor of TβRI would be introduced.

AZ12601011 and AZ12799734 are identified as two small molecule inhibitors of TβRI. They are found to be more effective than SB-431542 and galunisertib, the other two inhibitors of the receptor. AZ12601011 inhibits TβRI-mediated phosphorylation of Smad2 while AZ12799734 inhibits the TGF/BMP pathway. Thus, AZ12799734 not only suppresses the level of pSmad2 but also that of pSmad1 [160]. Both pSmad2 and pSmad1 are important mediators in the TGF-β pathway. Blocking the synthesis of pSmad2 and pSmad1 can impede the downstream cascade.

Galunisertib (LY2157299) is orally administered, lowering pSmad2 production in the TGF-β pathway by inhibiting TβRI. Clinical trials suggested that 150 mg galunisertib two times a day with durvalumab 1,500 mg Q4W as a combination therapy provides a period of tumor progression free survival in metastasizing pancreatic cancer [161]. It was found to be antineoplastic and radio-sensitizing, effective in hindering cancer cell migration in radiotherapy-resistant subjects with head and neck carcinoma (HNSCC). The anti-tumor effect is observed only in cell lines whose TGF-β pathway remains intact, which agrees with its role as a TβRI inhibitor [162]. In glioblastoma multiforme (GBM), NK cells cause the lysis of glioblastoma stem cells. However, the TGF-β pathway turns NK cells in the TME into an inhibitory phenotype, posing obstacles to the body’s natural immunity toward the tumor. Galunisertib is thus researched as a potential immunotherapeutic for GBM [163]. It also involves in the discussion on therapies for glioma and other advanced malignancies [164].

GFH018, another small molecule inhibitor of TβRI, has been under clinical investigation on its effectiveness as therapy of phase I/II advanced solid tumor [165]. Results have not yet been released but it was believed that the combined use with toripalimab, a monoclonal antibody targeting on PD-1 [166] would be useful in battling against the advanced solid tumor.

LY3200882 is a newly designed, selective adenosine triphosphate (ATP)–competitive inhibitor of the serine-threonine kinase domain of TβRI. It was proved to be a stronger inhibitor than Galunisertib preclinically [167]. The LY3200882 domain induces immunogenic cell death (ICD) of tumor and the maturation of DCs through its inhibition on TβRI. By interfering with the TGF-β pathway, the immunosuppressive TME is reversed, and immune cells can be revitalized. Studies had shown its possibile to be a drug for triple-negative breast cancer (TNBC) which is a highly recurrent, extensively metastasized malignancies with a few currently available treatments [168].

SB431542 promotes the non-anchoring growth of A549 cells and at the same time inhibits cell colonization in colon cancer which is suppressed and mediated by the TGF-β pathway respectively. It inhibits TβRI ALK4, 5, and 7. SB431542 blocks the formation of Smad2/3-Smad4 complexes and the translocation of Smad2/3 into the nucleus for transcription modification involved in the TGF-β pathway. The results of western blots further confirmed the reduction in end-protein expression of the pathway. However, it is noteworthy that SB431542 also inhibits TGF-β-mediated suppressive function on tumor such as the regulation of cell cycle and apoptosis so it should only be used when the tumor is unresponsive to these functions at first [169].

SD-208 downregulates paracrine and autocrine signaling of TGF-β in the LN308 human glioma sample. SD-208 was added to increase the ability of lymphocytes and T cells to lyse cancer cells. Their release of IFN-γ and tumor necrosis factor-alpha (TNF-α) were boosted as well. Although SD-208 can serve as both TβRI/II inhibitors, its specificity to TβRI is 100 folds more than that of the latter. The application of SD-208 seems not to affect cancer cell’s viability or proliferation but it is proven to slow down its progression and metastases [170]. Its use on the melanoma is also under investigation. As SD-208 reduces pSmad3 and inhibits Smads-specific transcription in the TGF-β pathway, it reduced the size of the osteolytic lesion due to bone metastases of melanoma in mice samples after 4 weeks of administration [171].

Vactosertib as known as TEW7197 was discovered as a specific TβRI inhibitor admitted orally. In a clinical trial regarding its effect on advanced solid tumors, it was found that no significant side effects have been caused in most patients but only fatigue [172]. As most other inhibitors mentioned, it can inhibit Smad2/3 activation in the TGF-β pathway. When used alone, it activates apoptosis in multiple myeloma (MM) cells and slows down its progression *in vitro*. Its use in combination with pomalidomide, an immunomodulatory drug used in MM is found to have augmented tumor suppressive effect on mice models [173]. The use of vactosertib with pembrolizumab on microsatellite stable metastatic colorectal cancer patients who were previously treated is also under investigation [174]. As a potent inhibitor, combination therapy in concern with vactosertib is being actively pursued in order to overcome the tumors’ resistance to other drugs available now.

YL-13027 is also a highly selective oral inhibitor of TβRI being used in phase I trial to test on its efficacy in treating the advanced solid tumor. It has a half-life of about 4.2 h on average. Unlike vactosertib, YL-13027 seems to induce adverse events (AEs) in a higher proportion of cancer subjects. It was reported that 12 out of 13 patients experienced AEs such as reduced hemoglobin (38.5%) and blood phosphorus (23.1%) [175].

Some small molecule inhibitors of TβRI were identified but are under research. PF-06952229 was found to have antineoplastic effects, especially in patients with metastasized breast cancer and solid tumors. Clinical trials are ongoing to investigate the pharmaceutical profile of the drug [176]. TD-1058’s use and mechanism were never published. The clinical trial on its use on idiopathic pulmonary fibrosis (IPF) was terminated [177, 178].

### Cancer vaccines

TGF-β is an essential regulator of the immunosuppressive state found in malignancies, and this has led to the creation of TGF-β specific vaccines. Tumor cell vaccines (TCVs) harness the antigen-presenting ability of immune cells and tumor antigens to enhance the body’s immune response against carcinoma. Tumor recurrence is also prevented due to the immunological memory of the immune cells. It is noted in much research that at the later stage of tumor progression, the tumor grows and metastasizes while bypassing the immunosurveillance. Thus, TCVs may only be an adjuvant in the early stage or residue disease [179].

Belagenpumatucel-L, an allogeneic TCV has been put into clinical trials to test its efficacy in treating advanced NSCLC and as a maintenance therapy for NSCLC [180, 181]. Belagenpumatucel-L consists of 4 TGF-β2-antisense gene-modified, irradiated, allogeneic NSCLC cell lines. Significant improvement in progression free survival by 12.4 months was observed in NSCLC patients receiving belagenpumatucel-L within 1 month after the termination of chemotherapy. It is noted that time after termination of initial therapy is critical in calculating the efficacy of belagenpumatucel-L as found that there is no obvious survival difference between patients receiving belagenpumatucel-L and placebo after a certain period [181].

Gemogenovatucel-T, as known as Vigil, is an autologous DNA transfected TCV. It provides anti-tumor effects by helping the immune system to recognize new foreign antigens and suppresses TGF-β1 and TGF-β2 levels by a furin-targeting RNA. At the intradermal injection site, the expression of granulocyte-macrophage colony-stimulating factor (GM-CSF), a cytokine responsible for the generation of DCs is promoted [182]. DCs are known to be one of the most potent antigen-presenting cells in the body. The use of Vigil in ovarian cancers was being studied. In a phase 2b trial, 3.1 months longer recurrence-free survival was recorded in the group of patients receiving Vigil instead of a placebo. Among the 47 patients receiving Vigil, only 3 experienced AEs with no grade 3 or 4 events reported [183].

Based on the comprehensive and detailed description above of the prospective options for targeted treatment on the TGF-β pathway, the Table 2 is summarized.

**Table 2.** Overview of TGF-β signaling immunotherapies

**Type**	**Treatment**	**Mechanism**	**Status**	**Reference**
Antibodies	Fresolimumab	Neutralization of TGF-β1, TGF-β2 and TGF-β3	Phase II	[124]
	NIS793	Neutralization of pan TGF-β ligands	Phase II	[125]
	SAR439459	Neutralization of TGF-β1, TGF-β2 and TGF-β3	Phase I/II	[126]
	TβM1 (LY2382770)	Selective neutralization of TGF-β1	Phase I	[127]
	XPA-42-089	Blockage of TGF-β1 and TGF-β2	Preclinical	[125]
	ABBV-151	Blockage of the GARP-TGF-β1 complex and the release of TGF-β1	Preclinical	[128]
	Anti-LAP	Reducing the latent release of inactive TGF-β from LAP/TGF-β complex	Phase I	[129]
	264RAD	Inhibition on αvβ6 and αvβ8 integrin	Preclinical	[130]
	Anti-αVβ8 (ADWA-11)	Neutralization of αVβ8	Preclinical	[131]
	Anti-αVβ8 (C6D4)	Neutralization of αVβ8	Preclinical	[132]
	SRK-181	Binding to latent TGF-β to prevent its activation	Phase I	[133]
	IMC-TR1 (LY3022859)	Inhibition on TβRII	Phase I	[134]
ASOs	ISTH0036	Selective inhibition on *TGF-β2* mRNA	Phase I	[140]
	ISTH0047 or ISTH1047	Reduction of TGF-β1 or TGF-β2 expression	Preclinical	[141]
	Trabedersen (AP 12009)	Inhibition on TGF-β2 expression	Phase I/II/III	[143]
Bifunctional fusion protein	4T-Trap	Binding to CD4 and inhibiting Th cell TGF-β signaling	Preclinical	[147]
	AVID200	Neutralization of TGF-β1 and TGF-β3	Phase I	[148]
	Bintrafusp alfa (M7824)	Reduction in expression of TGF-β and blockage of PD-L1 on CD8^+^ T cells	Phase II/III	[149]
	SHR-1701	Blockage of TGF-β/TβR pathway and rescue on PD-1/PD-L1 pathway	Phase I	[152]
	sTβRII-Fc	Inhibition on TGF-β1-dependent Smad3 phosphorylation	Preclinical	[154]
	Soluble β-glycan/TβRIII	Activating the TGF-β2-Smad signaling and the expression of TβRIII	Preclinical	[157]
Small-molecule inhibitors	AZ12601011, AZ12799734	Inhibition on TβRI-mediated phosphorylation of Smad2 and BMP pathway	Preclinical	[160]
	Galunisertib (LY2157299)	Inhibition on TβRI-mediated phosphorylation of Smad2	Phase I/II	[161]
	GFH-018	Inhibition on TβRI	Phase I/II	[165]
	LY3200882	Competitive inhibition on the serine-threonine kinase domain of TβRI	Phase I/II	[167]
	PF-06952229	Inhibition on TβRI	Phase I	[169]
	SB431542	Inhibition on TβRI	Preclinical	[171]
	SD-208	Inhibition on both TβRI and TβRII	Preclinical	[171]
	TD-1058	Inhibition on TβRI	Phase I	[178]
	Vactosertib (TEW7197)	Inhibition on TβRI	Phase I/II	[172]
	YL-13027	Inhibition on TβRI	Phase I	[175]
TCVs	Belagenpumatucel-L	Expressing the antisense strand of the TGF-β2 to suppress the expression of TGF-β2	Phase II/III	[181]
	Gemogenovatucel-T	Suppression on TGF-β1 and TGF-β2 levels by a furin-targeting RNA	Phase II	[182]

## Conclusion

TGF-β tumor-suppressive effects are often lost in advanced tumors, while tumor pro-invasive functions prevail. The increase in the secretion of TGF-β in the TME stimulates cancer cell growth, migration, and invasion. CAF induction, angiogenesis, and local immunosuppression are promoted, further contributing to tumor progression and metastasis. Several complementary pieces of evidence from preclinical and translational studies indicated that inhibition of the TGF-β/Smad3 pathway could be a potential cancer treatment. However, the transition from preclinical research to clinical practice has been unsuccessful and largely hindered. None of the TGF-β inhibitors evaluated in clinical trials was approved for cancer treatment because TGF-β is essential for several normal physiological processes in the human body, such as the gene deletion of TGF-β1, autoimmunity, abundant T cell proliferation and activation, and Th1 differentiation. The destruction of TGF-β1/Smad3 in the *in vivo* model leads to impaired immunity, indicating its importance in T cell development. In addition, inhibition of TGF-β signaling can lead to the development of keratoacanthoma (KA) and SCC by causing chronic inflammation of the skin and intestines [184, 185]. Several TβRI inhibitors promote the incidence of heart valve bleeding and its inflammatory or degenerative changes [186]. Although the combined use of TGF-β inhibitors and other checkpoint inhibitors is popular in the immunotherapy era, only some patients respond to the treatment. This highlights the need for the revaluation of laboratory data and analysis of the downstream modulators of the TGF-β pathway to identify potential therapeutic targets.

Thankfully, new research has shown that multiple unique TGF-β1/Smad3-dependent downstream regulators may control TGF-β1/Smad3 signaling without impacting its physiological activities. Src and Pou4f1 have been identified as important downstream regulators of TGF-β1/Smad3 signaling [8, 9]. These two transcription factors restore renal fibrosis via the signaling pathway and have high therapeutic potential. The miRNA and long non-coding RNA related to TGF-β1/Smad3 signaling are also under investigation. For example, upregulating the transcription of anti-fibrotic miR-200c and miR-141 can inhibit tumor cell metastasis and invasion [187, 188]. Besides, inhibition of Smad3-dependent long non-coding RNA erb-b2 receptor tyrosine kinase 4 intracellular domain fragment (Erbb4-IR) suppresses the proliferation of esophageal SCC by upregulating the tumor suppressor gene *miRNA-145* to activate cancer cells apoptosis [189]. In addition, the function of TGF-β1/Smad3 is highly dependent on the environment, and its tumor suppressive effect is reported in a cell-type-specific manner [190, 191]. The pathogenic effects of immune cells mediated through the TGF-β pathway in the TME, especially those mediated by the members of innate immunity such as macrophages, neutrophils, and DCs have been elucidated thoroughly in new cancer treatments [192]. These downstream goals may uncover new cancer treatment options.

In conclusion, a better understanding of the downstream signaling of TGF-β will reveal precise therapeutic targets in the microenvironment of cancer fibrosis, and further studies may determine their pathogenic roles in the TME as well as their therapeutic potential in cancer.

## Abbreviations

AEs: adverse events
AKT: protein kinase B
Bcl-2: B cell lymphoma-2
BMP: bone morphogenetic protein
CAF: cancer associated fibroblasts
CDK: cyclin-dependent kinase
Co-Smad: common-mediator mothers against decapentaplegic
CTGF: connective tissue growth factors
DCs: dendritic cells
EMT: epithelial mesenchymal transition
G1: Gap 1 phase
GTP: guanosine triphosphate
Id1: inhibitor of DNA binding 1
IFN-γ: interferon-γ
IgM: immunoglobulin M
JNK: c-Jun N-terminal kinase
LAP: latency associated peptide
MAPK: mitogen-activated protein kinase
MDSC: myeloid-derived suppressor cells
miR-301-3p: microRNA-301-3p
MKK3/6: mitogen-activated protein kinase kinase 3/6
mRNA: messenger RNA
mTOR: mammalian target of rapamycin
NK: natural killer
NSCLC: non-small-cell lung cancer
PD-1: programmed cell death protein 1
PD-L1: programmed cell death ligand 1
PI3K: phosphoinositide 3-kinase
pSmad2: phosphorylated mothers against decapentaplegic 2
RhoA: Ras homolog gene family member A
R-Smads: receptor-associated mothers against decapentaplegics
rTGF-β1: recombinant transforming growth factor-β
SCC: squamous cell carcinoma
ShcA: sarcoma homology 2 domain containing transforming protein A
Src: sarcoma
TAK1: transforming growth factor-activated kinase 1
TAM: tumor-associated macrophage
TCVs: tumor cell vaccines
TGF-β: transforming growth factor-β
TH17: T helper 17
TME: tumor microenvironment
TRAF6: tumor necrosis factor receptor-associated factor 6
Tregs: regulatory T cells
TβRI: transforming growth factor-β receptors I
Wnt: Wingless-related integration site
YAP: Yes-associated protein

## Declarations

### Copyright

© The Author(s) 2023.
